# Supplementary material for: Structural basis for antibacterial peptide self‐immunity by the bacterial ABC transporter McjD
Source: EMBO J. 2017 Sep 1;36(20):3062–79. doi: 10.15252/embj.201797278 (PMC5641919; doi:10.15252/embj.201797278)
Supplement: Supplementary file 1 — Expanded View Figures PDF [file EMBJ-36-3062-s001.pdf]

Expanded View Figures

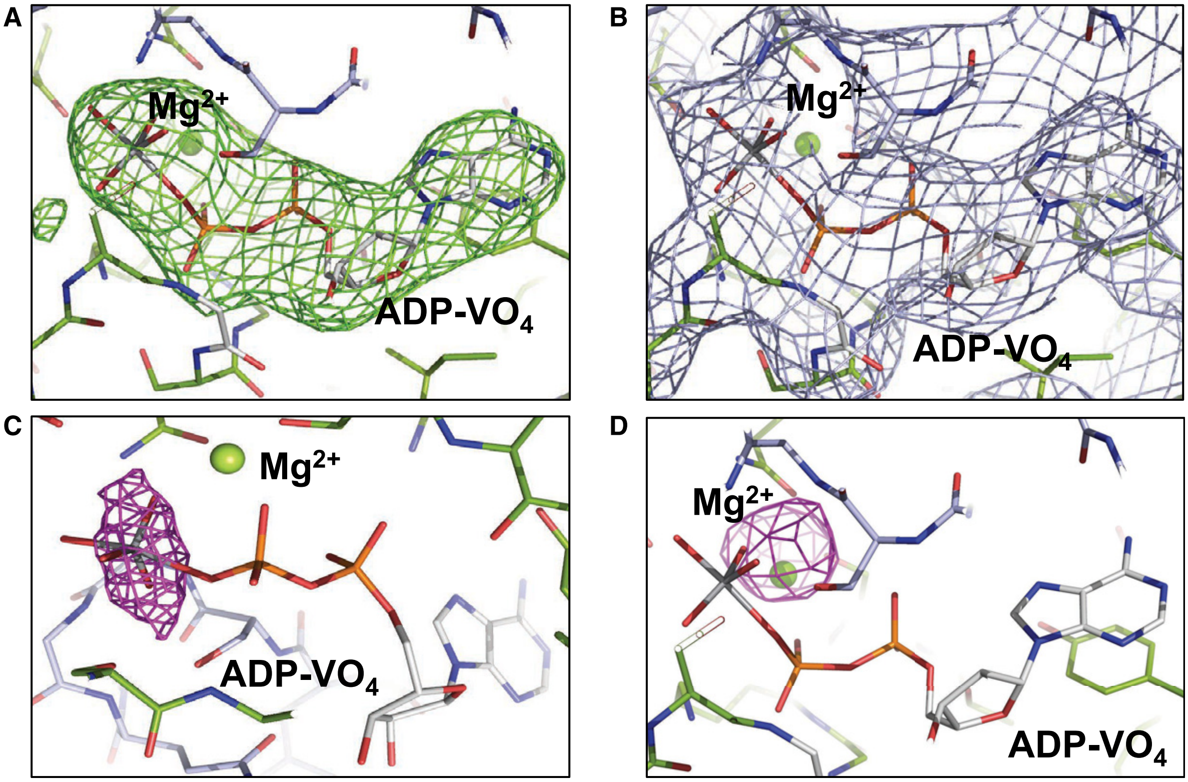

Figure EV1. Electron density maps.

A Clear |Fo|-|Fc| electron density map (green mesh contoured at 3 σ) could be observed around the ADV-VO_4 molecule after molecular replacement. ADV-VO_4 is only shown for clarity but it was not included in the refinement.

B Final 2|Fo|-|Fc| electron density map (blue mesh contoured at 1 σ) after including the ADV-VO_4 in the refinement. No negative or positive electron density peaks are observed.

C Anomalous difference electron density map (purple mesh) around the vanadate, from data collected close to the vanadium edge, 2.26 Å. The map is contoured at 10 σ. McjD and ADV-VO_4 are shown as sticks. The magnesium ion is shown as green sphere. McjD is coloured as in Fig 1. The ADV-VO_4 carbons are coloured grey, oxygens red, phosphate orange and vanadate dark grey. Colouring scheme as in Fig 1.

D Positive |Fo|-|Fc| electron density map (purple mesh contoured at 3 σ) around Mg^{2+} after excluding it from the refinement.

© 2017 The Authors

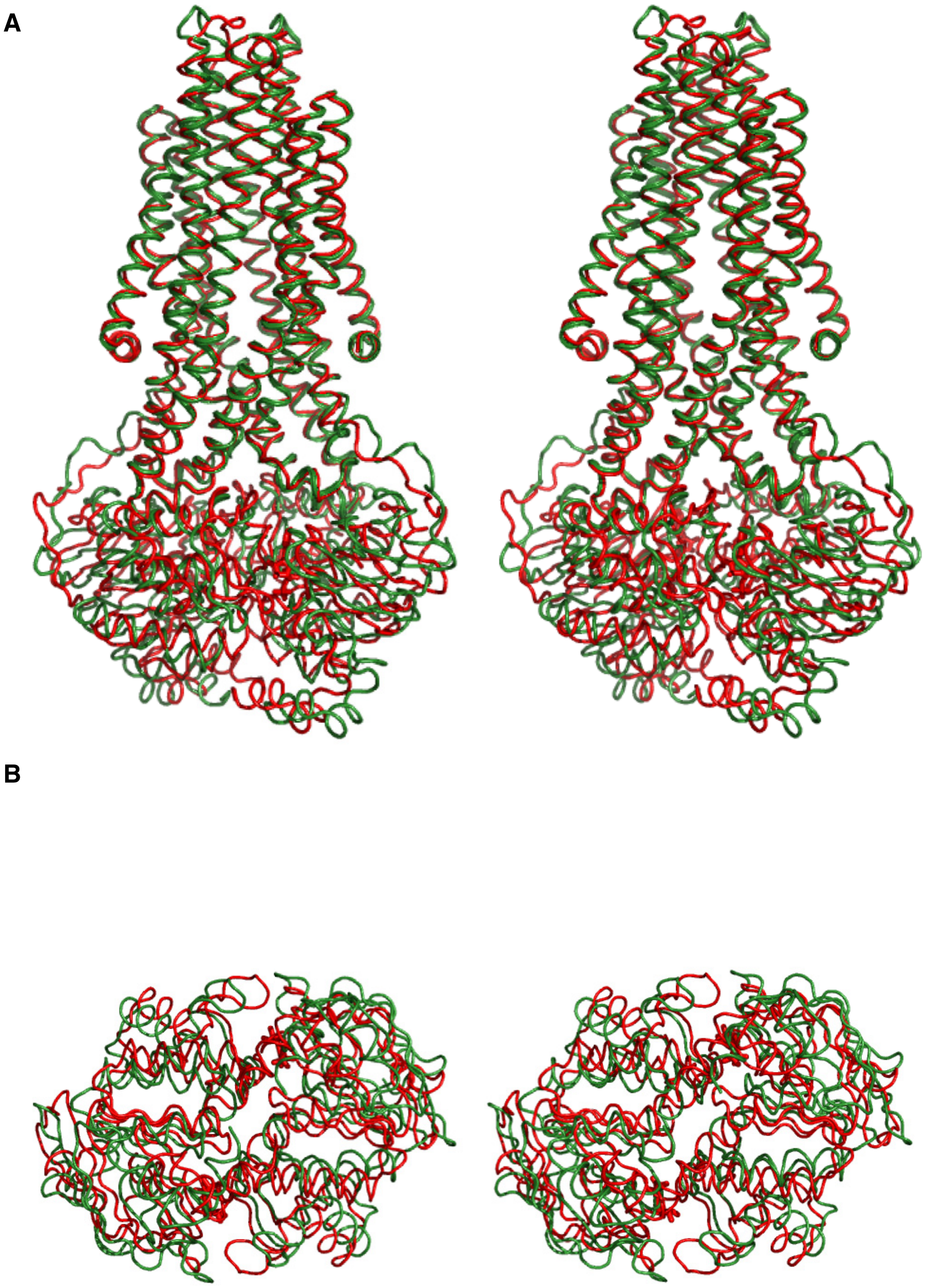

Figure EV2. Comparison of McjD-ADP-VO_4 and McjD-apo.

A Stereo figure of McjD-ADP-VO_4 (red ribbon) superimposed on McjD-apo (green ribbon) at the TMDs. View along the membrane. No significant changes are observed along the TMDs, whereas the NBDs do not align.

B Stereo figure of McjD-ADP-VO_4 (red ribbon) and McjD-apo (green ribbon) NBDs. View from the cytoplasmic side. The NBDs of the McjD-apo have disengaged in the absence of nucleotides.

 © 2017 The Authors

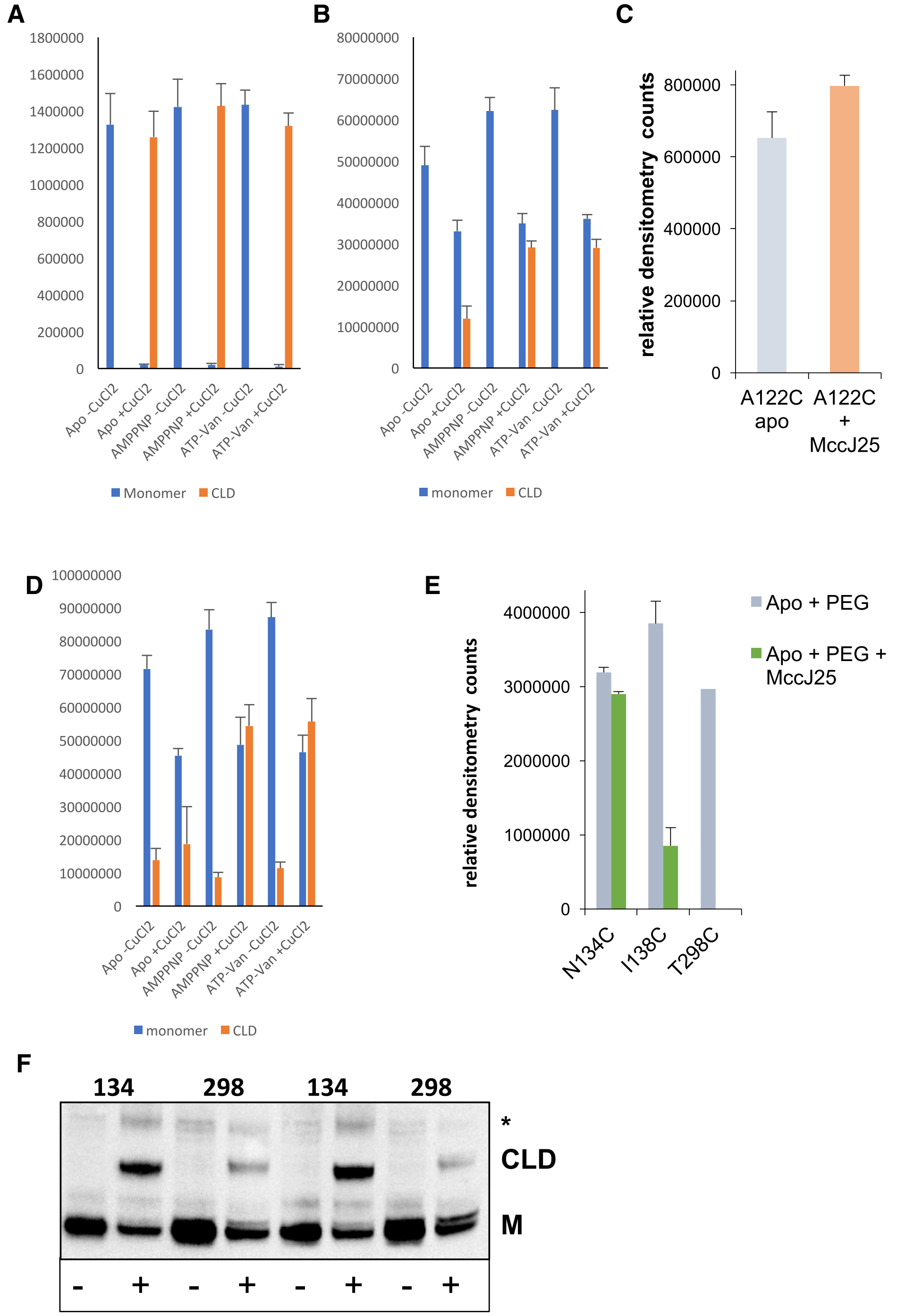

Figure EV3. Densitometry analysis of Western blots.

A Densitometry analysis of the L53C Western blot from Fig 4A.

B Densitometry analysis of the A122C Western blot from Fig 4B.

C The degree of cross-linking of McjD A122C in ISOVs in the absence and presence of MccJ25 was estimated by densitometry of the Western blot from Fig 4B (bottom panel). In the presence of MccJ25, a small enhancement of cross-linking was observed.

D Densitometry analysis of the S509C Western blot from Fig 4C.

E The degree of mPEG-10k to modify the cavity mutants in the absence and presence of MccJ25 was estimated by densitometry of the Western blot from Fig 7C. N134C can be PEGylated in the presence of MccJ25, whereas T298C shows abolishment of PEGylation. I138C shows very reduced PEGylation in the presence of MccJ25.

F The sampling of an inward-open conformation is not affected by pH. The PEGylation is as effective at pH 9 as at pH 7.5 (Fig 7), suggesting that the pH does not induce an inward-occluded conformation. Asterisk (*) denotes SDS-stable dimers.

Data information: Error bars are shown for all densitometry measurements (mean ± SEM; n = 3 with the exception of the competition assay that is n = 2).

© 2017 The Authors

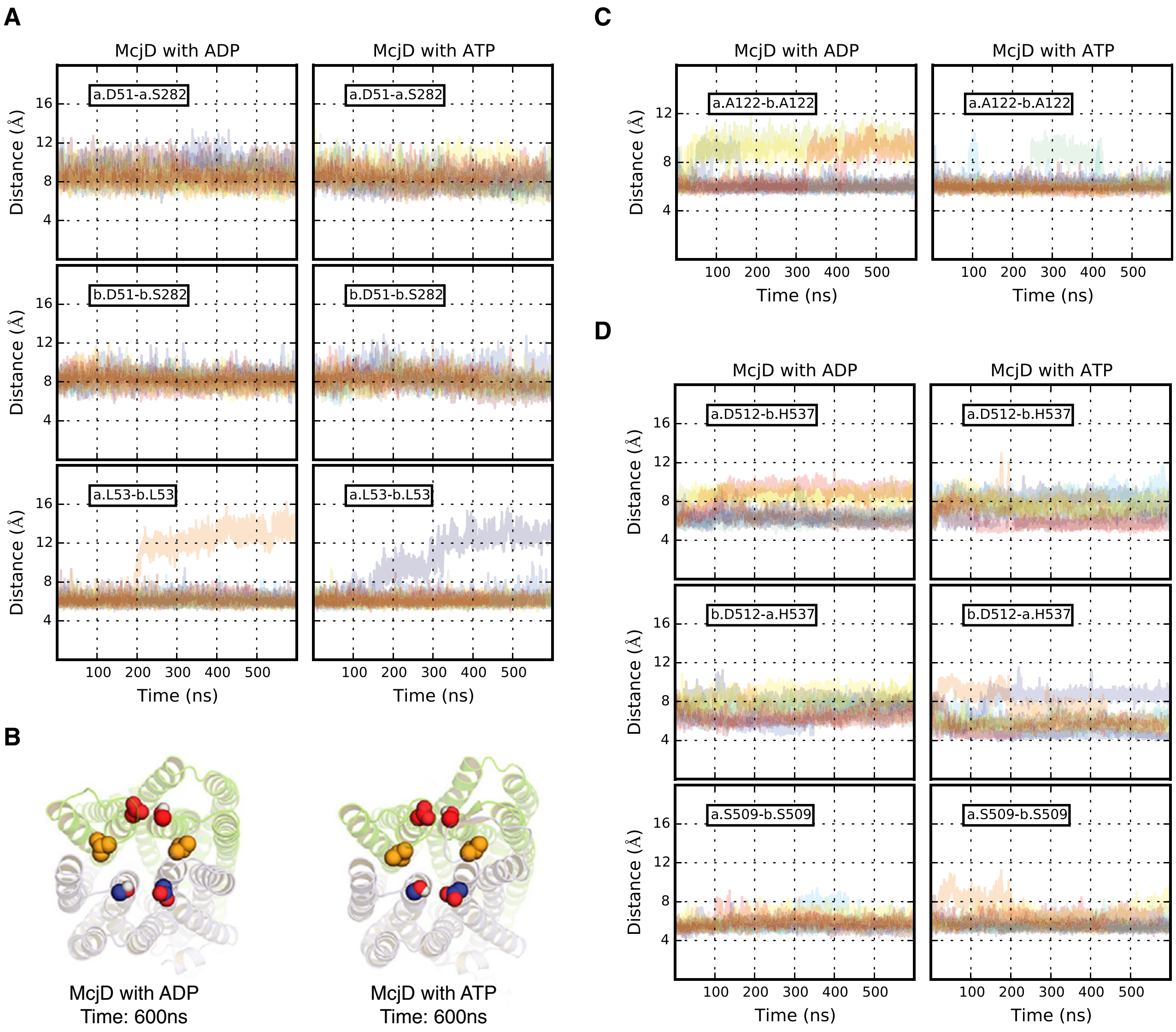

Figure EV4. MD simulations time profiles.

A–D Distances as a function of time for selected residues pairs in the (A) periplasmic loops, (C) cavity bottom and (D) NBDs interface. The time profiles of the 10 runs of each simulation set (ADP-bound, left, and ATP-bound, right) are shown in different colours. (B) Protein snapshots taken at 600 ns from the simulations with increased L53–L53 (yellow spheres) distance. Lipids and water are not shown for clarity. Colours and representation styles are as in Fig 8.

 © 2017 The Authors

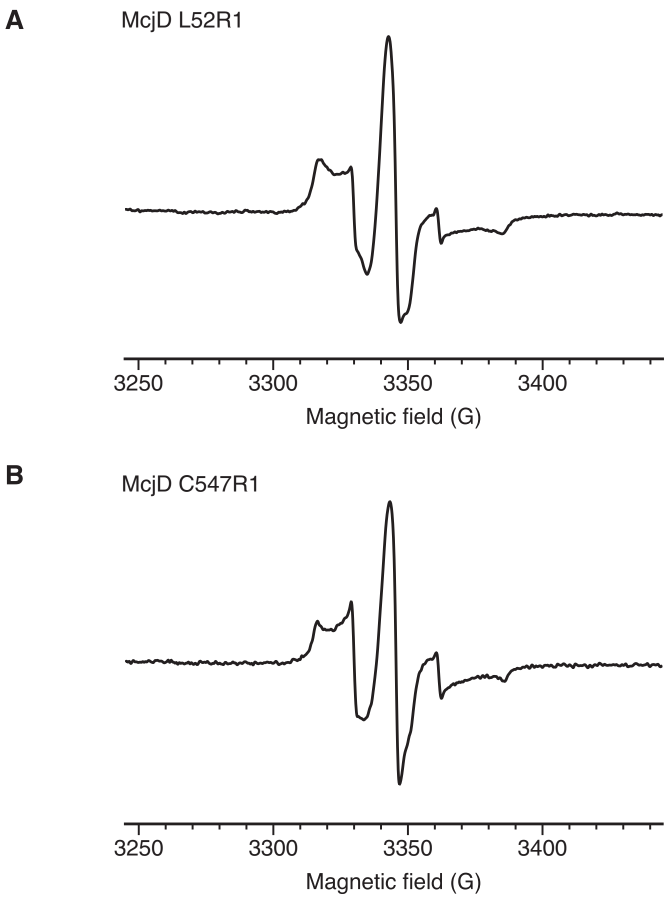

Figure EV5. *cw*-X-band EPR spectra.

A, B (A) McjD L52R1 and (B) McjD C547R1. EPR spectra were recorded on Bruker EMX micro X-band EPR spectrometer equipped with a super high-sensitivity (SHQ) resonator for X-band measurements. The samples were measured at room temperature with a microwave power of 20.0 mW, a video amplifier gain of 58 dB, a modulation amplitude of 1 G, a time constant of 40.96 ms and a conversion time of 42 ms.

© 2017 The Authors
