# Supplementary material for: Structural basis for antibacterial peptide self‐immunity by the bacterial ABC transporter McjD
Source: EMBO J. 2017 Sep 1;36(20):3062–79. doi: 10.15252/embj.201797278 (PMC5641919; doi:10.15252/embj.201797278)
Supplement: Supplementary file 3 — Source Data for Figure 4 [file EMBJ-36-3062-s002.pdf]

A

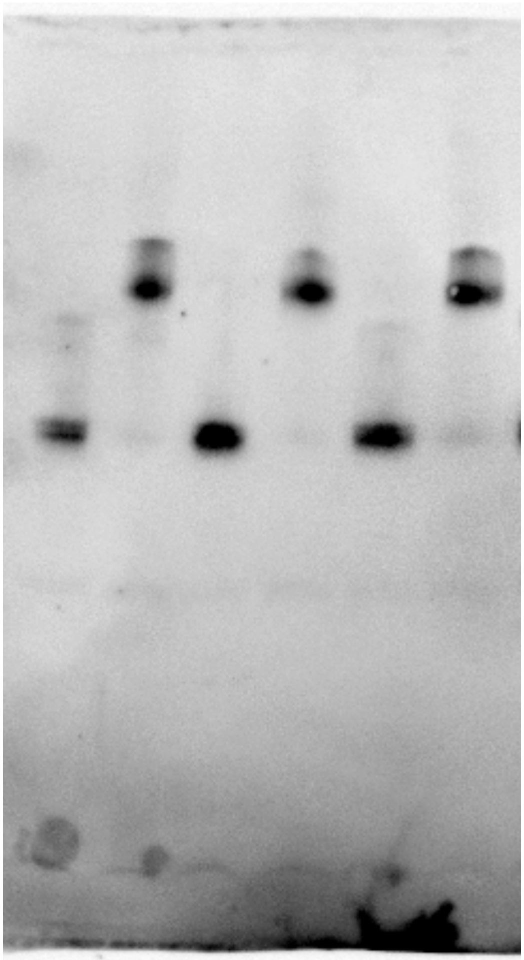

Uncropped Western blot related to Figure 4A

B

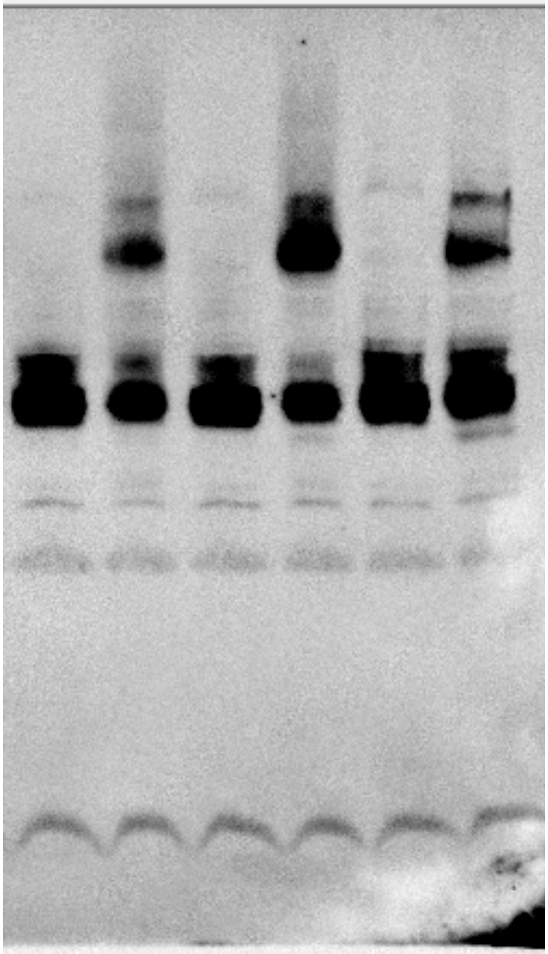

Uncropped Western blot related to Figure 4B (top panel)

C

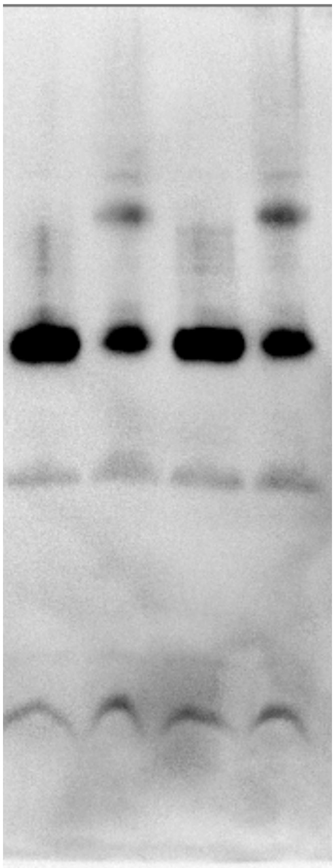

Uncropped Western blot related to Figure 4B (bottom panel)

D

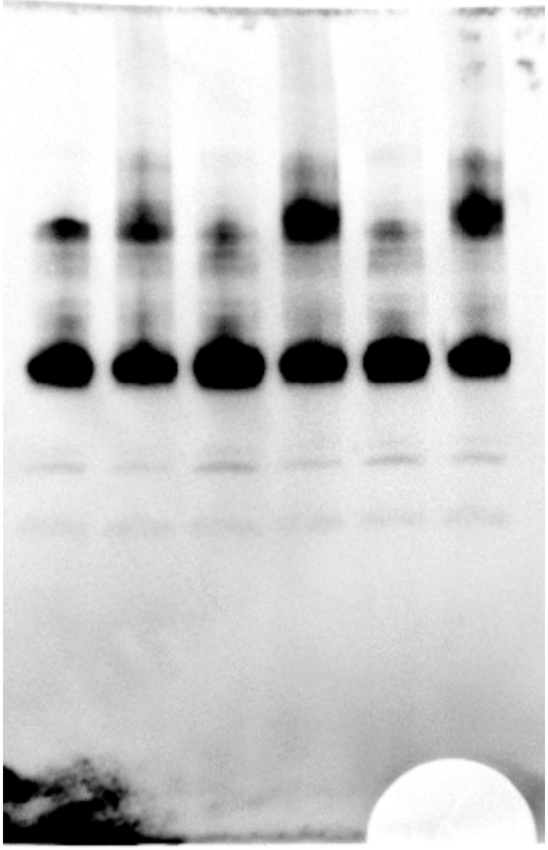

Uncropped Western blot related to Figure 4C
