# Supplementary figures and images for: Structural basis for antibacterial peptide self‐immunity by the bacterial ABC transporter McjD
Source: EMBO J. 2017 Sep 1;36(20):3062–79. doi: 10.15252/embj.201797278 (PMC5641919; doi:10.15252/embj.201797278)

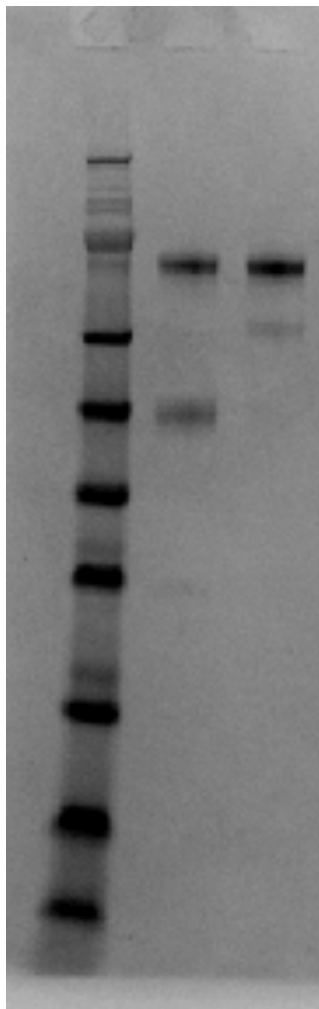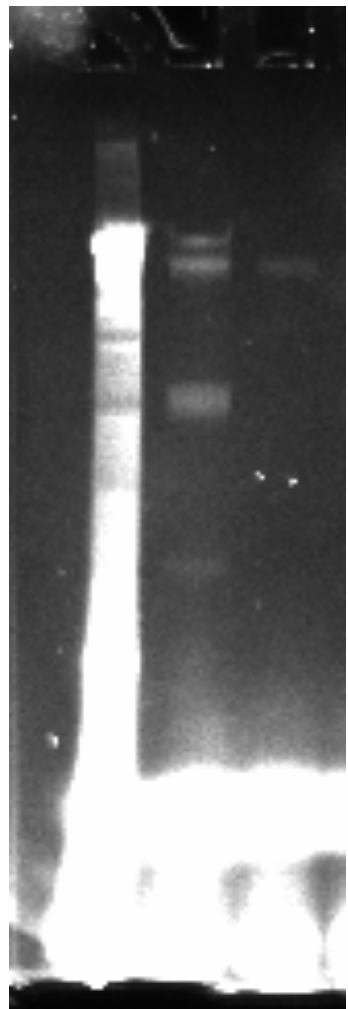

**Uncropped SDS-PAGE related to Figure 6D**

Supplement: Supplementary file 4 — Source Data for Figure 6 [file EMBJ-36-3062-s003.pdf]
